# Unveiling the LncRNA-miRNA-mRNA Regulatory Network in Arsenic-Induced Nerve Injury in Rats through High-Throughput Sequencing

**DOI:** 10.3390/toxics11120953

**Published:** 2023-11-22

**Authors:** Fang Chu, Chunqing Lu, Zhe Jiao, Wenjing Yang, Xiyue Yang, Hao Ma, Hao Yu, Sheng Wang, Yang Li, Dianjun Sun, Hongna Sun

**Affiliations:** 1 Institute for Endemic Fluorosis Control, Center for Endemic Disease Control, Chinese Center for Disease Control and Prevention, National Health Commission Key Laboratory of Etiology and Epidemiology, Harbin Medical University, Harbin 150081, China; chuf1996@163.com (F.C.); lcq2020@hrbmu.edu.cn (C.L.); wenjing0855@hotmail.com (W.Y.); yangxiyue1999@163.com (X.Y.); mh08080908@163.com (H.M.); m2011527395@163.com (H.Y.); 18347807511@163.com (S.W.); liyang_epi@163.com (Y.L.); 2 Heilongjiang Provincial Key Laboratory of Trace Elements and Human Health & Key Laboratory of Etiology and Epidemiology, Education Bureau of Heilongjiang Province, Harbin Medical University, Harbin 150081, China; hrbmujz@163.com; 3 Institute for Kashin-Beck Disease Control and Prevention, Chinese Center for Disease Control and Prevention, Harbin Medical University, Harbin 150081, China

**Keywords:** arsenic, nerve, high-throughput sequencing, ceRNA, lncRNA

## Abstract

Arsenic is a natural toxin which is widely distributed in the environment, incurring diverse toxicities and health problems. Previous studies have shown that long non-coding RNAs (LncRNAs) are also reported to contribute to As-induced adverse effects. LncRNAs are involved in the development of nerve injury, generally acting as sponges for microRNAs (miRNAs). This study aimed to investigate the competitive endogenous RNA (ceRNA) regulatory networks associated with arsenic-induced nerve damage. A total of 40 male Wistar rats were exposed to different doses of arsenic for 12 weeks, and samples were collected for pathological observation and high-throughput sequencing. The ceRNA network was constructed using Cytoscape, and key genes were identified through the PPI network and CytoHubba methods. A real-time quantitative PCR assay was performed to validate gene expression levels. The results showed that subchronic exposure to arsenic in drinking water resulted in pathological and ultrastructural damage to the hippocampal tissue, including changes in neuron morphology, mitochondria, and synapses. Exposure to arsenic results in the dysregulation of LncRNA and mRNA expression in the hippocampal tissues of rats. These molecules participated in multiple ceRNA axes and formed a network of ceRNAs associated with nerve injury. This study also verified key molecules within the ceRNA network and provided preliminary evidence implicating the ENRNOT-00000022622-miR-206-3p-Bdnf axis in the mechanism of neural damage induced by arsenic in rats. These findings provide novel insights into the underlying mechanism of nervous system damage induced by arsenic exposure.

## 1. Introduction

Arsenic, a naturally occurring non-metallic substance, is widespread in the environment and poses a significant health risk to humans [1]. Arsenic exposure primarily arises from the consumption of contaminated water, and it is estimated that a substantial population, ranging from 94 million to 220 million individuals, may encounter elevated levels of arsenic in groundwater [2]. Regions such as China [3], Bangladesh [4], and Latin America [5] are particularly affected, and even developed countries face potential risks [2]. Chronic arsenic exposure can lead to various diseases, including liver, skin, nervous system, and immune system disorders [6]. The neurological damage caused by arsenic exposure is of particular concern and requires comprehensive understanding to mitigate the associated health effects.

Recent research has highlighted the role of non-coding RNAs, including microRNAs (miRNAs) and long non-coding RNAs (lncRNAs), in arsenic-induced nerve damage [7]. LncRNAs are RNA molecules that do not encode proteins but play a crucial role in gene regulation, influencing various physiological processes [8]. They can interact with miRNAs as competitive endogenous RNAs (ceRNAs), modulating the regulation of target mRNAs by miRNAs [9]. This interaction forms complex regulatory networks involving multiple lncRNAs, miRNAs, and mRNAs, which are vital in physiological and pathological processes [10].

Although the ceRNA regulatory network has been extensively studied in various diseases [11,12,13], its specific role in arsenic-induced neuronal injury remains limited. Therefore, this study aims to investigate and explore the ceRNA regulatory network associated with arsenic-induced neurological damage using an experimental rat model exposed to arsenic. By elucidating the intricate interactions among lncRNAs, miRNAs, and mRNAs, this research aims to enhance our exploration of the underlying mechanism and potential therapeutic targets.

## 2. Materials and Methods

### 2.1. Experimental Animals and Grouping

A total of 40 male Wistar rats, aged 3 weeks and weighing 55 ± 10 g, were obtained from Beijing Viton Lihua Laboratory Animal Technology Co., Ltd., Beijing, China. The rats were housed in accordance with animal husbandry laws, ensuring full compliance. They were exposed to natural day and night lighting conditions, with an average indoor temperature of 22 ± 2 °C and a relative humidity of 40–60%. The rats had ad libitum access to food and water throughout the study. The experimental protocol was approved by the ethics committee of Harbin Medical University and adhered to the established animal ethics standards. After an initial period of one week for acclimation and regular feeding, the rats were randomly assigned to four groups based on their body weight using a random number table. The rats in the four groups were exposed to 0, 2, 10, and 50 mg/L sodium arsenite (NaAsO_2_, CAS No. 7784-46-5, purchased from Beijing InnoChem Science & Technology Co., Ltd., Beijing, China) solutions in their drinking water for 12 weeks, respectively. Water intake for each group was recorded throughout the 12-week arsenic exposure period. Detailed information on water intake and arsenic levels can be found in Supplemental Table S1 and Supplemental Table S2, respectively.

### 2.2. Main Reagents and Instruments

We used sodium arsenite (Harbin Medical University), hematoxylin staining solution (Biosharp, Hefei, China), eosin staining solution (Biosharp, Hefei, China), xylene (Tianjin Bodi Chemical Co., Ltd., Tianjin, China), anhydrous ethanol (Tianjin Fuyu Fine Chemical Co., Ltd., Tianjin, China), paraformaldehyde (Tianjin Bodi Chemical Co., Ltd., Tianjin, China), concentrated sulfuric acid (Chengdu Sitiande Biological Co., Ltd., Chengdu, China), concentrated nitric acid (Chengdu Sitiande Biological Co., Ltd., Chengdu, China), perchloric acid (Shanghai Chemical Reagent No. 2 Factory, Shanghai, China), thiourea (KASMA Technology Co., Ltd., Hong Kong), ascorbic acid (KASMA Technology Co., Ltd., Hong Kong), potassium borohydride (Shanghai Chemical Reagent No. 2 Factory, Shanghai, China), arsenic standard solution (CDC Nutrition Institute, Beijing, China), a reverse transcription kit (Takara, Osaka, Japan), and a real-time fluorescence quantitative PCR kit (Takara, Osaka, Japan). We also used a biological tissue dehydrator (Hubei Taiwei Medical Technology Co., Ltd., Xiaogan, China), biological tissue embedding instrument (Hubei Taiwei Medical Technology Co., Ltd., Xiaogan, China), optical microscope (Olympus Optical Industries, Tokyo, Japan), thermostatic digestion instrument (Qiqihar Precision Instrument Factory, Qiqihar, China), atomic fluorescence spectrometer (Beijing Jitian Instruments Co., Ltd., Beijing, China), gradient PCR instrument (ABI, Waltham City, MA, USA), and real-time quantitative PCR instrument (ABI, Waltham City, MA, USA). Finally, we utilized a PCR instrument (ABI, Waltham City, MA, USA).

### 2.3. Execution of Experimental Animals and Sample Collection

After 12 weeks of arsenic exposure, the rats were euthanized using an intraperitoneal injection of a combination of 10% chloral hydrate and alfentanil hydrochloride. Subsequently, the rats were swiftly dissected, and the brain tissue was carefully isolated. The brain tissue was then divided into two parts: seven pieces from the left hemisphere were fixed in paraformaldehyde for further processing, while three pieces of the hippocampus were carefully excised and fixed in glutaraldehyde and subsequently stored at a temperature of 4 °C away from light. The hippocampal tissue obtained from the other hemisphere was promptly preserved in liquid nitrogen using sterilized frozen storage tubes. Following this, the samples were transferred to a storage temperature of −80 °C for long-term preservation.

### 2.4. Determination of Arsenic Content

The samples of urine, serum, and brain tissue were collected and processed following the most recent Chinese national standard method (GB/T 5750.6-2006) [14,15]. Subsequently, the atomic fluorescence intensity of the samples was measured using hydride atomic fluorescence spectrophotometry. The fluorescence intensity was linearly associated with the quantity of arsenic ions throughout the range of the standard curve, allowing the arsenic content of the samples to be determined.

### 2.5. Brain Histopathology Section and Nissl Staining

The paraffin-embedded rat brain tissues were fixed, rinsed, dehydrated, made translucent, and then sectioned at a thickness of 5 μm. The sections were subjected to a routine dewaxing process to remove the paraffin. Nissl staining was performed by immersing the sections in a Nissl staining solution for 50 min at a temperature of 55 °C. After staining, the sections were carefully removed and rinsed three times with distilled water. Subsequently, a standard step-by-step dehydration process was conducted, followed by drying. Finally, the sections were sealed with neutral gum. The morphology of nerve cells and Nissl body was observed using a light microscope.

### 2.6. Transmission Electron Microscopy

Transmission electron microscopy (TEM) was employed to observe the ultrastructure of neurons in the hippocampal tissue. First, the tissue was fixed in glutaraldehyde for 2 h and sectioned into small 1 mm^3^ pieces. These sections were then subjected to a second fixation with 4% glutaraldehyde, followed by cleaning with PBS and fixation with 1% osmic acid. Afterward, the tissue underwent gradient dehydration using an acetone solution. Subsequently, the tissue was embedded in Epon812 embedding agent and polymerized in an incubator. Semi-thin sections were selected, and ultrathin sections were prepared. These sections were then double stained with uranium acetate and lead citrate to enhance the contrast. Finally, the stained sections were observed under a transmission electron microscope to examine the ultrastructure of the neurons.

### 2.7. High-Throughput Sequencing

High-throughput sequencing was conducted to analyze the hippocampal tissues of three randomly selected rats from both the control and the high-dose arsenic-exposed group. High-throughput sequencing was completed by Novogene Co., Ltd. (Beijing, China). The operation procedure is briefly described as follows: After total RNA extraction, the Ribo-ZeroTM rRNA Removal Kit was used to remove rRNA and achieve RNA fragmentation (average fragment length of approximately 200 nt). Single-stranded cDNA was synthesized using reverse transcription, and then double-stranded cDNA was synthesized. After the purification of double-stranded cDNA, terminal repair was performed. The sample was added with primers, amplified, and purified through PCR for library construction according to RNA species. After the quality inspection of the library, samples were sequenced using the Illumina HiSeqTM 2500 sequencing platform. After obtaining the raw data through sequencing, an information analysis process was performed. Compared to the control group, the “DESeq2” package in R software 4.1.2 as used to identify the differentially expressed mRNAs and lncRNAs with thresholds of |fold change (FC)| > 1.5 and adjusted *p*-value <  0.05. The “ClusterProfiler” package in R software was used for functional enrichment analysis, and GO biological processes and KEGG pathways at the significant level (q-value < 0.05) were employed.

### 2.8. Construction of Regulatory Network

The construction of the regulatory network was based on the ceRNA theory, which suggests that lncRNAs and mRNAs compete for binding to miRNAs when they share miRNA binding sites. To identify target miRNA molecules of differentially expressed lncRNAs and mRNAs, a combination of co-localization and co-expression techniques was employed.

For differential lncRNAs, target miRNA molecules were predicted using the miRanda database based on their co-localization and co-expression patterns. For differential mRNAs, target miRNA molecules were predicted using online databases such as TargetScan, miRDB, and miRWalk. The intersection of miRNA molecules from these three databases was considered as the target miRNA molecules for differential mRNAs.

Next, the target miRNA molecules of differential lncRNAs were intersected with the target miRNA molecules of differential mRNAs. The resulting miRNA molecules, along with their corresponding target lncRNAs and mRNAs, were used to construct the ceRNA network. The network was visualized using the Cytoscape software (version 3.7.0, Cytoscape consortium) [16], where different shapes represented various RNA types, and different colors indicated the up- or down-regulation of the RNA expression levels. This visualization facilitated the interpretation of the regulatory interactions between lncRNAs, mRNAs, and miRNAs in the ceRNA network.

### 2.9. Analysis of Network Key Nodes

To analyze the key nodes in the network, PPI (Protein–Protein Interaction) network analysis was conducted using the online database STRING (https://cn.string-db.org/, accessed on 16 April 2022). The network was visualized using Cytoscape software.

To identify the critical genes in the network, the Cytohubba plug-in, which is integrated into Cytoscape, was utilized. Cytohubba calculates three centrality metrics for each node in the network: degree centrality, betweenness centrality, and closeness centrality. Degree centrality measures the number of connections a node has with other nodes in the network. Betweenness centrality quantifies the extent to which a node acts as a bridge between other nodes in the network. Closeness centrality reflects the proximity between a node and other nodes in the network. The top 10 molecules that appeared in the intersection of the three centrality metrics were considered as important node molecules in the network.

### 2.10. Real-Time Quantitative PCR

RNA was extracted using the Trizol method, with six samples in each group. For mRNA and lncRNA detection, cDNA was synthesized through reverse transcription using a reverse transcription kit from Takara (RR047A). For miRNA detection, a transcription kit from Takara (638315) was used. The reaction conditions for the cDNA synthesis methods of lncRNA and mRNA were: 15 min at 37 °C, followed by 5 s at 85 °C. The reaction conditions for the cDNA synthesis methods of miRNA were: 60 min at 37 °C, followed by 5 s at 85 °C. The qPCR reactions were performed using a quantitative PCR kit from Takara (RR820A). The PCR reaction system consisted of 5.0 µL of SYBR, 0.2 µL of ROX Reference Dye II, 0.4 µL each of forward and reverse primers, 3 µL of DEPC water, and 1 µL of cDNA. The reaction conditions were as follows: pre-denaturation at 95 °C for 10 min, denaturation at 95 °C for 15 s, annealing at 60 °C for 1 min, and a total of 40 cycles. The internal reference genes used in the analysis were actin and U6. Two secondary wells were set up for each sample to ensure accuracy. The expression level of the target gene was determined by calculating the 2^−∆∆^CT value.

### 2.11. Statistical Analysis

Statistical analysis was performed using SPSS 22.0 software. Values are presented as mean ± SD unless indicated otherwise. A one-way ANOVA was used to compare the differences between groups, and further two-by-two comparisons were made using the LSD method or Dunnett’s T3 test. Statistical significance was selected at *p* < 0.05.

## 3. Results

### 3.1. Effects of Arsenic Exposure on Arsenic Levels in Rats

**Figure 1:**
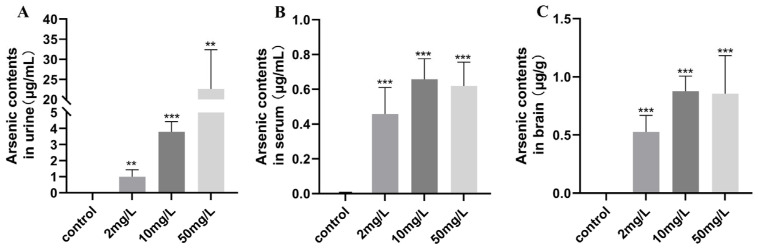
Arsenic levels in rats. (**A**) Arsenic contents in urine. (**B**) Arsenic contents in serum. (**C**) Arsenic contents in brain. Values are the mean ± SD. ** *p* < 0.01; *** *p* < 0.001, compared with the control group, *n* = 10.

This study investigated the effects of subchronic drinking water arsenic exposure on arsenic levels in rats. The results showed that the arsenic-exposed rats had significantly higher levels of arsenic in urine, serum, and brain tissue compared to the control group (*p* < 0.05, as shown in Figure 1A–C).

### 3.2. Morphological Damage to Hippocampal Neurons in Arsenic-Exposed Rats

**Figure 2:**
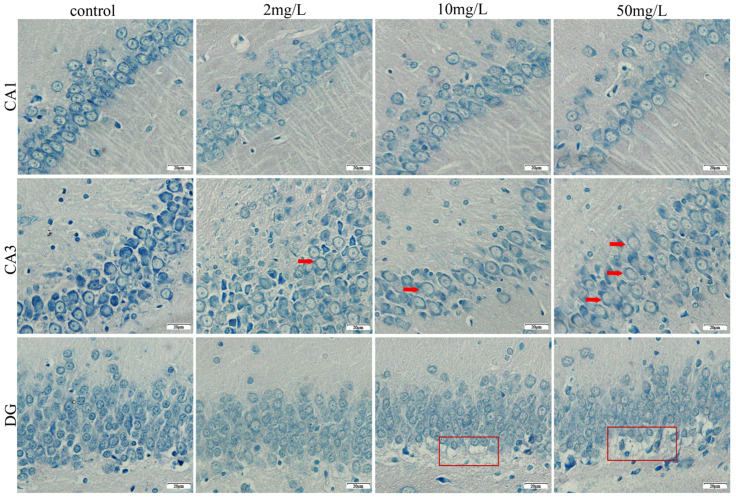
Nissl staining of rat hippocampus (magnification, 400×). Red arrow: nucleolus disappears. Red frame: vacuolization of cells.

Nissl staining revealed significant neuronal damage in the hippocampus of arsenic-exposed groups. Representative images (Figure 2) showed that the neurons in the control group displayed an intact structure and organized arrangement, with dark blue cytoplasm and abundant Nissl bodies. In contrast, the neurons of the arsenic-exposed group showed lighter cytoplasm staining, reduced Nissl body content, and varying degrees of cell damage in the CA1, CA3, and DG regions. As arsenic exposure increased, the number of cell layers decreased in the CA1 region, accompanied by progressive cell disorganization. In the CA3 region, low-dose arsenic exposure induced cell proliferation, while medium- and high-dose arsenic exposure led to decreased cell count and irregular cell morphology. Arsenic-exposed groups exhibited vacuolization of underlying cells in the DG region, with deeper vacuolization observed in the high-dose arsenic-exposed group. These findings confirm that arsenic exposure causes morphological damage to hippocampal neurons in rats.

### 3.3. Ultrastructural Damage of Hippocampal Neurons in Arsenic-Exposed Rats

**Figure 3:**
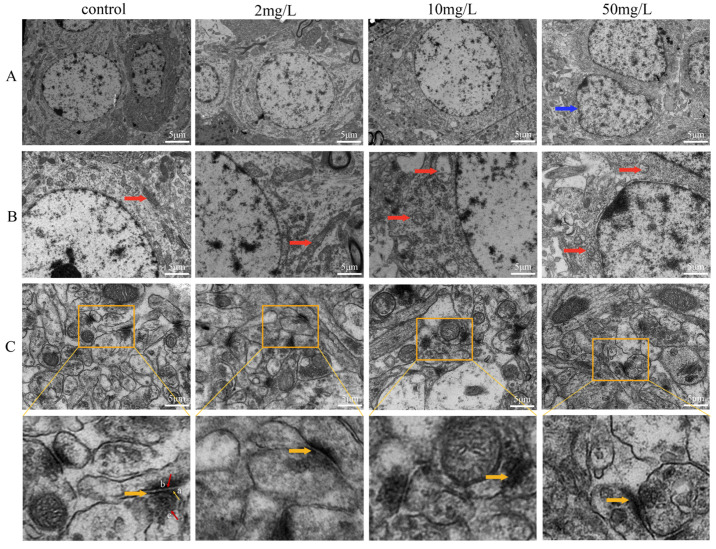
Electron microscopic observation of hippocampal neurons in rats (magnification, 1000×). (**A**) Nucleus. Blue arrow: nuclear envelope depression. (**B**) Organelles. Red arrow: mitochondria. (**C**) Synapses. Yellow squares represent the selected parts of the enlarged image. Yellow arrow: Enlarged synapses. a: Pre-synaptic membrane. b: Post-synaptic membrane. c: Synaptic vesicle.

Transmission electron microscopy was used to investigate the effects of arsenic exposure on the ultrastructure of hippocampal neurons. In the control group, hippocampal neurons exhibited well-defined nuclear membrane boundaries, round nuclei, and deep cytoplasmic staining. However, in the high-dose arsenic-exposed group, the nuclei displayed irregular shapes and slight indentations (Figure 3A). Mitochondria exhibited the most significant alterations among the organelles, characterized by pronounced swelling in all arsenic-exposed groups and reduced or disappeared mitochondrial ridges in the medium- and high-dose arsenic-exposed group (Figure 3B). The control group showed intact structure of the presynaptic membrane and the posterior membrane, a clear synaptic cleft, and abundant synaptic vesicles. However, with an increasing concentration of arsenic exposure, the boundaries of the synaptic structures became blurred, and the number of synaptic vesicles decreased (Figure 3C).

### 3.4. Arsenic Exposure Induces RNA Expression Differences in Rat Hippocampus

**Figure 4:**
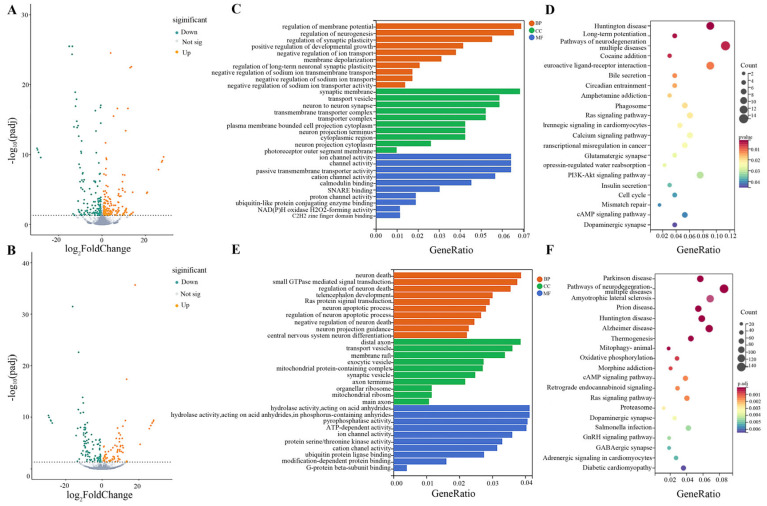
Results of high−throughput sequencing. (**A**) Volcano plot of differentially expressed mRNAs. (**B**) Volcano plot of differentially expressed lncRNAs. (**C**) GO function analysis of differential expressed mRNAs. BP: biological process; CC: cellular components; MF: molecular function. (**D**) Top 20 KEGG pathways of differentially expressed mRNAs. (**E**) GO function analysis of differential expressed lncRNAs. BP: biological process; CC: cellular components; MF: molecular function. (**F**) Top 20 KEGG pathways of differentially expressed lncRNAs.

Differential gene expression analysis was conducted on rat hippocampal tissue to assess the impact of subchronic drinking water arsenic exposure. According to a prespecified threshold (|FoldChange| > 1.5, adjusted *p*-value < 0.05), a total of 388 DE mRNAs, including 203 up-regulated and 185 down-regulated (Figure 4A), 177 DE lncRNA, including 75 up-regulated and 102 down-regulated (Figure 4B), were obtained in the high-dose arsenic-exposed group compared to the control group.

### 3.5. Functional Enrichment of GO and KEGG for Differential mRNA Molecules

To gain insights into the functional implications of the differential mRNA molecules between the high-dose arsenic-exposed group and the control group, GO enrichment analysis and KEGG pathway analysis were performed. GO enrichment analysis revealed enrichment in ion transport, synaptic plasticity, and neurogenesis in biological processes. In cellular components, enrichment was observed in synaptic membranes, neuronal projection cytoplasm, and transporter protein complexes. Molecular functions were predominantly enriched in ion channel activity, transporter activity, and ubiquitin-like protein-binding enzyme binding (Figure 4C). KEGG pathway analysis was conducted to identify the relevant pathways associated with the differential mRNA molecules. The analysis revealed the involvement of pathways such as Huntington disease, long-term potentiation, and pathways of neurodegeneration multiple diseases (Figure 4D). These findings provide insights into the functional implications of arsenic exposure on the molecular processes and pathways associated with brain function.

### 3.6. Functional Enrichment of GO and KEGG for Differential lncRNA Molecules

Functional enrichment analysis was performed on the differential lncRNA molecules between the high-dose arsenic-exposed group and the control group. GO enrichment analysis revealed enrichment in biological processes such as neuronal differentiation, regulation of neuronal death, and neuronal death in the CNS. In terms of cellular components, enrichment was observed in mitochondrial protein-containing complexes, distal axons, and organellar ribosomes. Molecular function analysis indicated enrichment in ion channel activity, hydrolase activity, and ATP-dependent activity (Figure 4E). KEGG pathway analysis identified Parkinson’s disease, pathways of neurodegeneration multiple diseases, and amyotrophic lateral sclerosis as significant pathways (Figure 4F). These results provide insights into the functional implications of arsenic exposure on lncRNA-associated processes and pathways in the rat brain.

### 3.7. Construction of Lnc-mi-mRNA Regulatory Network

**Figure 5:**
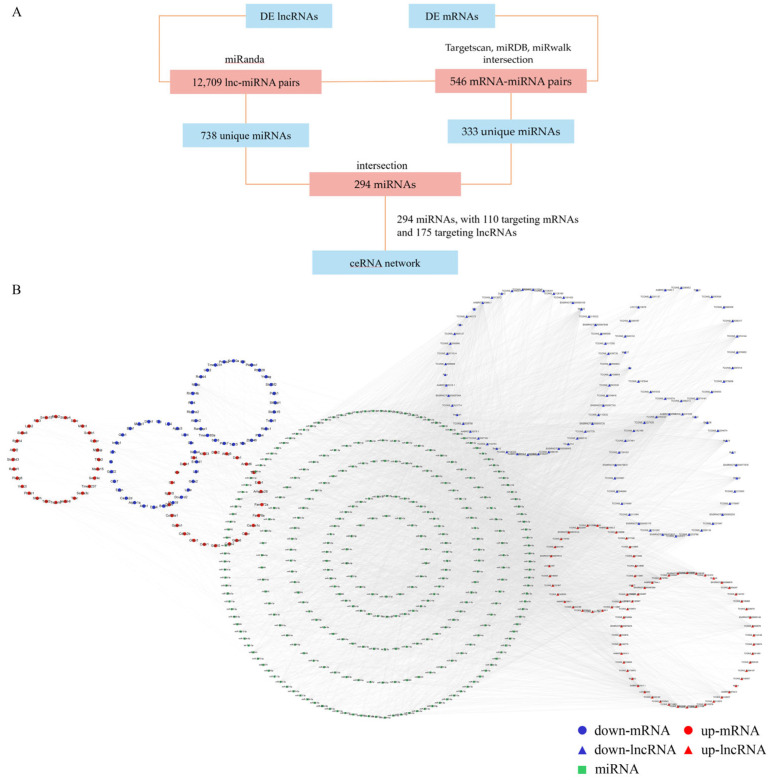
(**A**) Flow chart of lnc-mi-mRNA regulatory network construction. (**B**) lncRNA-miRNA-mRNA interaction network. Red and blue represent up-regulated and down-regulated RNAs, respectively. Squares, circles, and triangles represent miRNAs, mRNAs, and lncRNAs, respectively.

A lnc-mi-mRNA regulatory network was constructed to investigate the interactions between differentially expressed lncRNAs and mRNAs. The lnc-mi-mRNA regulatory network was constructed as the flow chart which is shown in Figure 5A. Initially, 12,709 lnc-miRNA pairs were predicted using miRanda, resulting in 738 unique miRNAs. Subsequently, the databases Targetscan, miRDB, and miRwalk identified 546 mRNA-miRNA pairs, encompassing 333 unique miRNAs. Intersection analysis revealed 294 miRNAs that targeted both differential lncRNAs and mRNAs. The resulting ceRNA network included 294 miRNAs, 110 targeted mRNAs, and 175 lncRNAs. The network was visualized in Cytoscape 3.7.0 (Figure 5B). This network provides a view of the regulatory interactions between lncRNAs, miRNAs, and mRNAs in response to arsenic exposure in rats’ hippocampus.

### 3.8. Screening of Key mRNA in the Lnc-mi-mRNA Regulatory Network

**Figure 6:**
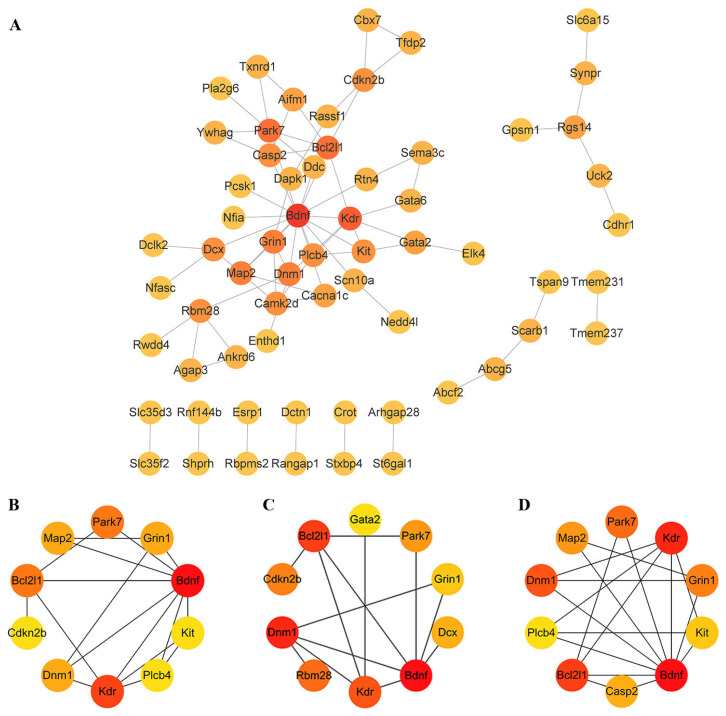
Screening of key mRNA in the lnc-mi-mRNA regulatory network. (**A**) A protein–protein interaction network constructed based on 110 differential mRNA molecules. (**B**) The top 10 molecules identified with degree algorithm. (**C**) The top 10 molecules identified with betweenness algorithm. (**D**) The top 10 molecules identified with closeness algorithm. The shade of the color represents the degree of criticality, with a closer resemblance to red indicating a higher level of criticality for the molecule.

To identify key nodes in the lnc-mi-mRNA regulatory network, the 110 differential mRNA molecules were subjected to protein–protein interaction (PPI) network analysis. This analysis resulted in the inclusion of 63 molecules and 73 interaction edges (Figure 6A). Three algorithms, namely degree, betweenness, and closeness, were utilized to identify the top 10 molecules based on their network properties (Figure 6B–D). The intersection of the molecules identified by these three methods led to the selection of Grin1, Dnm1, Bcl2l1, Bdnf, Park7, and Kdr as key molecules. Among these key molecules, Bdnf, Grin1, Park7, and Bcl2l1 were considered critical for further validation as enriched in the neurodegeneration multiple illness pathways, highlighting their potential role in disease processes. Further investigation and validation of these molecules could provide valuable insights into the underlying mechanisms influenced by the lncRNA-mediated regulatory network.

### 3.9. Validation of ceRNA Regulatory Networks

#### 3.9.1. Validation Results of mRNA

**Figure 7:**
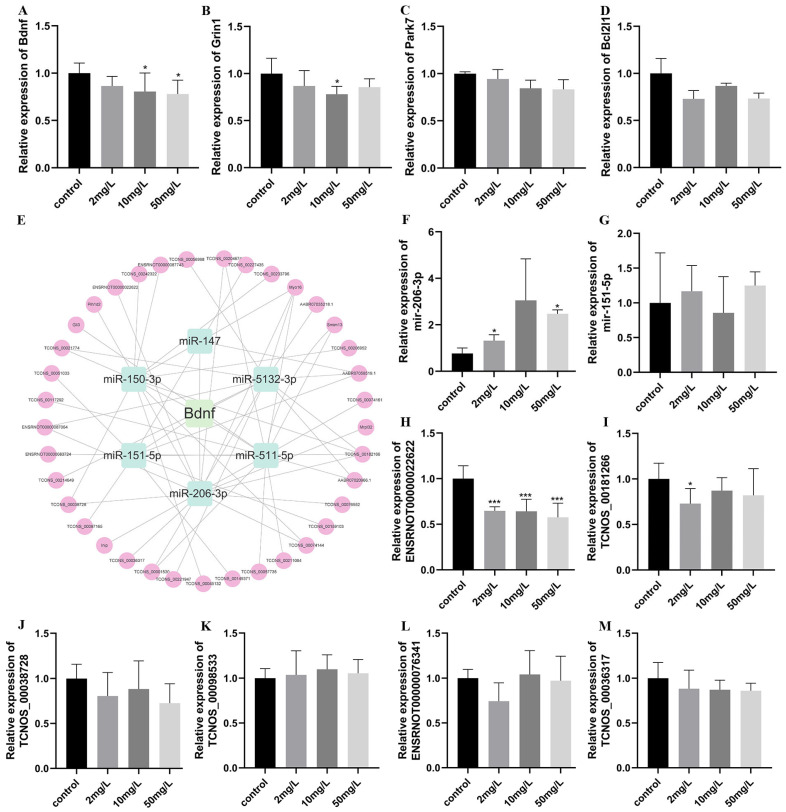
Validation of ceRNA regulatory network. (**A**–**D**) Relative expression of mRNA (Bdnf, Grin1, Park7, Bcl2l1). (**E**) A ceRNA network with Bdnf as the axis. The green squares around Bdnf represent miRNA; the pink circles represent the lncRNA. (**F**–**G**) Relative expression of miRNA (miR-206-3p, miR-151-5p). (**H**–**M**) Relative expression of lncRNA. (ENSRNOT00000022622, TCNOS-00181266, TCNOS-00038728, TCNOS-00098533, ENSRNOT00000076341, TCNOS-00036317). Values are the mean ± SD, * *p* < 0.05, *** *p* < 0.001, compared with the control group, *n* = 6.

The expression levels of Bdnf, Grin1, Park7, and Bcl2l1 were assessed using real-time quantitative PCR in hippocampal tissues from six randomly selected rats in each group. Compared to the control group, all four molecules showed a declining trend in the arsenic-exposed groups. However, statistical analysis revealed that only the expressions of Bdnf and Grin1 exhibited significant differences (Figure 7A–D). Specifically, the expression of Bdnf showed the most significant decrease, and a dose-dependent relationship was observed. Accordingly, a ceRNA network diagram was constructed with Bdnf as the central axis (Figure 7E).

#### 3.9.2. Validation Results of miRNA

From the ceRNA network constructed with Bdnf as the central axis, six miRNAs with a high association strength were identified. Among them, the scores of miR-206-3p and miR-151-5p in the miRDB database were both higher than 80, and the sum of their scores in the targetscan database was smaller (low scores represent better-targeting relationships). This suggested a potential targeting relationship between Bdnf and miR-206-3p, as well as miR-151-5p. To further investigate, the expression levels of miR-206-3p and miR-151-5p were measured. The results revealed a significant upregulation of miR-206-3p in the arsenic-exposed group, with a statistically significant difference (*p* < 0.05, Figure 7F,G).

#### 3.9.3. Validation Results of lncRNA

There were 11 lncRNA molecules in total with a targeting connection to miR-206-3p and exhibiting an opposite expression trend. However, only six of them were expressed in the hippocampus above the detection threshold. We examined the expression levels of these six molecules, and the results indicated that among them, lncRNA-ENSRNOT-00022622 showed significant downregulation in the arsenic-exposed groups, with statistically significant differences compared to the control group (*p* < 0.001). However, the remaining five molecules did not exhibit statistically significant differences between the arsenic-exposed group and the control group (Figure 7H–M). These findings shed light on the regulatory mechanisms involving mRNA, miRNA, and lncRNA in response to arsenic exposure, particularly highlighting the role of Bdnf and its potential interactions with miR-206-3p and lncRNA-ENSRNOT-00022622.

## 4. Discussion

In this study, we aimed to gain an understanding of the effects of subchronic arsenic exposure on hippocampal neurons and organelles, as well as the expression profiles of lncRNAs, miRNAs, and mRNAs. By employing pathology techniques and transmission electron microscopy, we meticulously observed the morphological changes induced by arsenic in hippocampal neurons and organelles. High-throughput transcriptome sequencing provided valuable insights into the expression patterns of these non-coding and coding RNA molecules in the hippocampal tissues of arsenic-exposed rats. Through in-depth analysis of the interactions among these molecules, we constructed a potential ceRNA regulatory network and successfully validated one of the pathways.

Arsenic accumulates in the body with toxic effects mainly through drinking water. The World Health Organization (WHO) has established a guideline concentration of 10 μg/L for arsenic in drinking water. However, the problem of high levels of arsenic in drinking water remains a significant concern around the world. For instance, in regions like Bangladesh where arsenic levels in drinking water are high, the maximum exposure dose is reported to be 4.73 mg/L [17]. Considering the conversion based on body surface area, this human equivalent dose corresponds to approximately 27.91 mg/L in rats, falling within the range of the highest (50 mg/L) and medium (10 mg/L) doses used in our study. In addition, we used ad libitum drinking, allowing animals unrestricted access to water to simulate natural behavior and minimize potential biases. We observed a decrease in daily water intake with higher arsenic exposure, which may be attributed to taste aversion or physiological responses. However, the actual intake of arsenic increases with increasing exposure concentrations, leading to the accumulation of arsenic.

Assessing the concentration of arsenic in biological samples serves as an indicator of the body’s arsenic load. In our study, we observed an increase in arsenic levels in urine, serum, and brain samples as the arsenic concentration increased. Notably, there was no significant difference in the arsenic content between the middle-dose arsenic group and the high-dose arsenic group in serum and brain samples. Previous research has demonstrated that arsenic levels in biological samples tend to increase with higher arsenic concentrations. However, when the arsenic concentration increased exponentially, the corresponding change in arsenic levels in the blood–brain barrier did not exhibit an exponential pattern [18]. This observation is consistent with the results of our study and suggests that the brain may have a limited capacity to accumulate arsenic. This limited accumulation ability could be attributed to the brain’s mechanisms of arsenic absorption and regulation, which warrant further investigation.

The hippocampus, a critical brain region responsible for cognitive functions, including learning, memory, and spatial navigation [19], exhibits a high density of nerve cells and an active metabolism, rendering it particularly susceptible to the detrimental effects of toxic substances. Its distinct and well-defined structure, along with clear boundaries delineating various subregions, makes the hippocampal region an ideal target for investigating the structural and functional alterations induced by toxins [20]. Through the examination of these changes, valuable insights into the effects of poisons on the nervous system can be gleaned, providing important clues to elucidate the underlying mechanisms involved.

Nissl bodies are distinct, basophilic structures observed within the cytoplasm of neurons. They are composed of clusters of rough endoplasmic reticulum and ribosomes, which play a crucial role in protein synthesis [21,22]. Nissl bodies are abundant and large in neurons under healthy physiological conditions. However, in instances of neuronal damage or pathology, alterations in Nissl bodies can occur [23]. This can manifest as a reduction in or even the complete disappearance of Nissl bodies within affected neurons [24]. The loss or depletion of Nissl bodies indicates disruption in neuronal protein synthesis and overall cellular health. This is often associated with neuronal degeneration [25], dysfunction [26], or death [27]. Consistent with previous studies, our results showed a reduction in Nissl bodies in the arsenic-exposed groups, as indicated by the lighter Nissl staining. This reduction serves as an indicator of nervous system injury resulting from arsenic exposure. Furthermore, Nissl staining allowed us to observe pathological lesions in different brain regions. In the CA1 region, we observed a decrease in the number of vertebral cells following arsenic exposure. This finding suggests a potential link between arsenic exposure and cognitive abnormalities, such as decreased long-term memory. In the CA3 region, we observed abnormal cell proliferation or reduction after arsenic exposure. These changes in cell morphology and organization may impact the formation and retrieval of spatial memory, a function associated with the CA3 region [28]. Severe vacuolar degeneration was observed in the DG region. This vacuolar degeneration may be associated with nerve damage resembling depressive symptoms [29,30]. However, the underlying mechanisms of this association require further investigation.

Transmission electron microscopy revealed that high-dose arsenic exposure led to damage in the nucleus morphology of hippocampal neurons, while mitochondrial swelling and abnormal synaptic morphology were observed across all the arsenic exposure groups. Arsenic-induced mitochondrial swelling can disrupt energy production processes within the mitochondria, resulting in energy metabolism disorders [31]. Additionally, this may interfere with the antioxidant system of mitochondria, leading to elevated oxidative stress [32]. Furthermore, arsenic can interfere with the function of calcium channels and transporters in mitochondria, causing a disturbance in calcium balance [33]. The abnormal synaptic morphology induced by arsenic can disrupt normal neuronal signaling [34,35]. A decrease in the synaptic vesicle count or impairment of synaptic function can affect neurotransmitter release and the excitatory or inhibitory state of postsynaptic neurons [36]. Changes in structure may be accompanied by changes in function. Such an abnormal synaptic structure may heighten the risk of neurodegenerative diseases, including Alzheimer’s disease and Parkinson’s disease [37,38]. Further investigations are necessary to explore the underlying mechanisms responsible for these pathological injuries.

LncRNAs have garnered significant attention due to their ability to regulate gene expression indirectly. Extensive researches have illuminated the vital roles that lncRNAs played in neural development, brain function injury, and neurodegenerative changes [39]. These molecules are recognized as key regulators of neuronal differentiation, development, and regeneration within the intricate landscape of the nervous system [40]. This study also placed a significant focus on lncRNAs. Leveraging high-throughput sequencing techniques, we identified a total of 177 differentially expressed lncRNA molecules, with 75 lncRNAs being up-regulated and 102 lncRNAs being down-regulated. The KEGG functional enrichment analysis of differentially expressed lncRNAs uncovered their significant enrichment in signaling pathways related to neurodegeneration, Huntington’s disease, and other neurological injuries. Further analysis through GO indicated that these molecules are primarily associated with neuronal differentiation, altered ribosome function, and mitochondrial dysfunction. It is worth noting that the functional roles of many lncRNAs remain poorly understood, highlighting the need for further exploratory studies in this area.

The ceRNA hypothesis has unveiled novel mechanisms of RNA interaction [41]. According to this hypothesis, miRNAs possess the ability to bind to target mRNAs, thereby inhibiting their translation or directly triggering mRNA degradation. As a result, the post-transcriptional regulation of gene expression is achieved [42]. In addition, lncRNAs can serve as miRNA sponges, effectively inhibiting the activity of miRNAs by binding to miRNA recognition elements. This interaction indirectly influences mRNA expression [43]. The creation of ceRNA networks enables a deeper comprehension of the functions of unknown lncRNAs through the knowledge of known mRNAs. In our study, we constructed a ceRNA interaction network by integrating differential expression data and online databases. By applying various topological methods and conducting real-time quantitative PCR experiments, we identified Bdnf as a possible crucial molecule involved in injury within the constructed ceRNA network.

Bdnf is a member of the neurotrophic protein family, which plays a critical role in neuronal survival, the formation of new synapses, synaptic plasticity, dendritic branching, and the regulation of excitatory and inhibitory properties and neurotransmitters [44,45]. Bdnf plays a significant role in various stages of development and aging processes [46]. Decreased levels of Bdnf mRNA and protein have been observed in brain structures that are closely associated with cognitive function, such as the hippocampus and frontal cortex. Studies have found that reductions in Bdnf are linked to cognitive deficits that can ultimately lead to the development of Alzheimer’s disease and dementia [47]. Porritt et al. demonstrated that the local inhibition of Bdnf production caused a significant reduction in the function of dopaminergic neurons in the rat substantia nigra [48]. The overexpression of Bdnf can also induce an inflammatory response mediated by NF-κB. This leads to the activation of astrocytes, increased production of cytokines and neurotoxic reactive oxygen species, as well as the aggregation of activated microglia [49]. Bdnf exhibits the ability to bind to Trkb receptors and induce the phosphorylation of Trkb. This phosphorylation event triggers the activation of multiple signaling cascades, leading to an increase in synaptic plasticity and the modulation of neurogenesis. In conclusion, the dysregulation of Bdnf is known to play a crucial role in nerve damage. Furthermore, studies have revealed that miR-206-3p, a small RNA molecule, is implicated in neurological impairments including Alzheimer’s disease, depression, and neuropathic pain [50,51]. Research findings have provided evidence for the regulatory role of miR-206-3p as an upstream regulator of Bdnf. The inhibition of miR-206-3p has been shown to lead to the increased expression of Bdnf [52]. Our study discovered a novel hetero-lncRNA molecule, lncRNA-ENSRNOT00022622, through sequencing analysis. Our findings revealed a decreasing trend in the expression of lncRNA-ENSRNOT00022622, which aligns with the ceRNA hypothesis. Moving forward, our research will focus on investigating the potential role of lncRNA-ENSRNOT00022622 in arsenic-induced nerve damage. We aim to determine whether this lncRNA competes with Bdnf for binding to miR-206-3p, resulting in a decrease in Bdnf expression levels.

## 5. Conclusions

Subchronic exposure to arsenic in drinking water can cause pathological damage in hippocampal tissue, resulting in ultrastructural changes in hippocampal mitochondria and synapses. Subchronic arsenic exposure leads to the altered expression of multiple LncRNA and mRNA molecules in the rat hippocampus. These differentially expressed molecules are involved in multiple ceRNA axes, forming a ceRNA network associated with nerve damage. In addition, our study preliminarily verified that arsenic may be involved in the mechanism of nerve injury in rats through the ENSRNOT00000022622-miR-206-3p-Bdnf axis.

## Data Availability

The data presented in this study are available on request from the corresponding author.

## Supplementary Materials

The following supporting information can be downloaded at: https://www.mdpi.com/article/10.3390/toxics11120953/s1, Table S1: Water intake of rats. Table S2: Arsenic intake of rats.
