# Supplementary material for: Unveiling the LncRNA-miRNA-mRNA Regulatory Network in Arsenic-Induced Nerve Injury in Rats through High-Throughput Sequencing
Source: Toxics. 2023 Nov 22;11(12):953. doi: 10.3390/toxics11120953 (PMC10747658; doi:10.3390/toxics11120953)
Supplement: Supplementary file 1 [file toxics-11-00953-s001.zip › toxics-2700918-supplementary.pdf]

Table S1. Water intake of rats (ml, n=10).

	control	2mg/L	10mg/L	50mg/L
week1	2444.88	2366.17	2219.22	1919.01
week2	3261.16	3304.09	2981.78	2353.45
week3	3534.25	3692.43	2857.22	2046.87
week4	4121.40	3802.10	2967.00	1978.20
week5	5104.94	4448.77	3567.11	2071.61
week6	4819.30	4091.80	3250.20	1941.74
week7	5030.60	4549.90	3456.31	2005.40
week8	5084.46	4702.84	3500.40	1889.36
week9	5445.80	5340.00	3798.80	2323.20
week10	5224.06	4516.80	3752.90	1831.84
week11	5721.09	5606.40	4411.20	2387.86
week12	6122.32	4149.90	4507.08	2049.23
total	55914.26	50571.20	41269.22	24797.77

Table S2. Arsenic intake of rats (mg, n=10).

	control	2mg/L	10mg/L	50mg/L
week1	0.00	4.73	22.19	95.95
week2	0.00	6.61	29.82	117.67
week3	0.00	7.38	28.57	102.34
week4	0.00	7.60	29.67	98.91
week5	0.00	8.90	35.67	103.58
week6	0.00	8.18	32.50	97.09
week7	0.00	9.10	34.56	100.27
week8	0.00	9.41	35.00	94.47
week9	0.00	10.68	37.99	116.16
week10	0.00	9.03	37.53	91.59
week11	0.00	11.21	44.11	119.39
week12	0.00	8.30	45.07	102.46
total	0.00	101.14	412.69	1239.89
